# Outcome after Interdisciplinary Treatment for Aneurysmal Subarachnoid Hemorrhage—A Single Center Experience

**DOI:** 10.3390/medicina55110724

**Published:** 2019-11-01

**Authors:** Benjamin Voellger, Rosita Rupa, Christian Arndt, Barbara Carl, Christopher Nimsky

**Affiliations:** 1 Department of Neurosurgery, University Hospital Marburg, 35033 Marburg, Germany; rupar@med.uni-marburg.de (R.R.); carlb@med.uni-marburg.de (B.C.); nimsky@med.uni-marburg.de (C.N.); 2 Department of Anaesthesiology, University Hospital Marburg, 35033 Marburg, Germany; arndtc@med.uni-marburg.de

**Keywords:** aneurysmal subarachnoid hemorrhage, outcome, interdisciplinary setting

## Abstract

*Background and Objectives:* To identify predictors of outcome after aneurysmal subarachnoid hemorrhage (aSAH) in our interdisciplinary setting. *Materials and Methods:* 176 patients who had been treated for aSAH by a team of neurosurgeons and neuroradiologists between 2009 and 2017 were analyzed retrospectively. Age, gender, clinical presentation according to the Hunt and Hess (H&H) grading on admission, overall clot burden, aneurysm localization, modality of aneurysm obliteration, early deterioration (ED), occurrence of vasospasm in transcranial Doppler ultrasonography, delayed cerebral ischemia (DCI), spasmolysis, decompressive craniectomy (DC), cerebrospinal fluid (CSF) shunt placement, deep vein thrombosis (DVT), pulmonary embolism (PE), severe cardiac events (SCE), mortality on Days 14, and 30 after admission, and outcome at one year after the hemorrhage according to the Glasgow Outcome Scale (GOS) were recorded. Chi square, Fisher’s exact, Welch’s t, and Wilcoxon rank sum served as statistical tests. Generalized linear models were fitted, and ordered logistic regression was performed. *Results*: SCE (*p* = 0.049) were a significant predictor of mortality at 14 days after aSAH, but not later during the first year after the hemorrhage. Clipping as opposed to coiling (*p* = 0.049) of ruptured aneurysms was a significant predictor of survival on Day 30 after aSAH, but not later during the first year after the hemorrhage, while coiling as opposed to clipping of ruptured aneurysms was significantly related to a lower frequency of DVT during hospitalization (*p* = 0.024). Aneurysms of the anterior circulation were significantly more often clipped, while aneurysms of the posterior circulation were significantly more often coiled (*p* < 0.001). Age over 70 years (*p* = 0.049), H&H grade on admission (*p* = 0.022), overall clot burden (*p* = 0.035), ED (*p* = 0.009), DCI (*p* = 0.013), DC (*p* = 0.0005), and CSF shunt placement (*p* = 0.038) proved to be predictive of long-term outcome after aSAH. *Conclusion:* Long-term results after clipping and coiling of ruptured aneurysms appear equal in an interdisciplinary setting that takes aneurysm localization, available staff, and equipment into account.

## 1. Introduction

The high-level evidence provided by the International Subarachnoid Aneurysm Trial (ISAT) [1] led to favor coiling, a technique first described by Guglielmi [2], over clipping for the obliteration of ruptured cerebral aneurysms. Nonetheless, interdisciplinary settings were tailored to the local particularities at many neurovascular centers over time, due to the indisputable weaknesses of ISAT, namely the disproportionately high number of coiled aneurysms of the anterior communicating artery, and the comparison of the results of aneurysm obliteration performed by highly specialized neuroradiologists with those performed by averagely trained neurosurgeons in ISAT. The Barrow Ruptured Aneurysm Trial (BRAT) [3] initially seemed to confirm the results of ISAT, while data from the BRAT study at six years after the hemorrhage [4] suggested that the modality of aneurysm obliteration of ruptured aneurysms of the anterior circulation would not affect long-term results as long as treatment remained in experienced hands. Furthermore, data from the BRAT study at 10 years after the hemorrhage demonstrated that rates of complete aneurysm obliteration and rates of retreatment actually favored clipping over coiling [5].

There is, however, only a limited number of publications on results after single-center interdisciplinary treatment for aSAH. Güresir et al. [6] identified intraparenchymal hemorrhage as prognostically unfavorable in 585 patients treated interdisciplinarily for aSAH. Proust et al. [7] found a decrease of verbal memory capabilities in 50 patients after clipping versus coiling of ruptured aneurysms of the anterior communicating artery, while other neuropsychological deficiencies and quality of life did not differ significantly between treatment groups. The same group [8] reported on the results of interdisciplinary treatment at six months after aSAH, according to the modified Rankin Scale (mRS) [9] in 64 patients who were 70 years of age or older at the time of bleeding; an unfavorable correlation of initially poor clinical presentation and delayed cerebral ischemia with outcome was found. Schöller et al. [10] described an initially good clinical presentation and an age of less than 70 years on admission as prognostically favorable factors. In a small series of patients with ruptured aneurysms of the posterior inferior cerebellar artery, Sejkorova et al. [11] identified an initially high Hunt and Hess (H&H) [12] grade as unfavorable for the outcome after interdisciplinary treatment. Schwartz et al. [13] found young age at the time of admission and absence of cerebral ischemia to yield a favorable prognosis in 106 cases of interdisciplinary treated ruptured cerebral aneurysms. AlMatter et al. [14] identified age, initial clinical presentation, re-rupture of the aneurysm, intraparenchymal hemorrhage, and ruptured aneurysms of the middle cerebral artery as relevant prognostic factors; they described a trend towards unfavorable outcomes after vasospasm, intraventricular hemorrhage, and rupture of large aneurysms.

In our study, we aimed to retrospectively identify predictive factors after treatment for aSAH in a single center series of 176 cases.

## 2. Materials and Methods

### 2.1. Patients

In this single center retrospective study, 176 patients admitted to our university hospital between 2009 and 2017 were included. The patients fulfilled each of the following inclusion criteria: subarachnoid hemorrhage diagnosed after cranial computed tomography (CCT) or lumbar puncture, detection of at least one cerebral aneurysm in digital subtraction angiography (DSA) or computed tomography angiography (CTA), obliteration of the ruptured aneurysm by coiling or clipping within 24 h after admission. Patients with a history of severe cognitive impairment prior to the hemorrhage, such as progressive dementia, and patients with H&H grade 5 hemorrhages who did not benefit from external ventricular drainage (EVD) insertion were not included.

Age, gender, aneurysm localization, blood distribution according to the modified Fisher scale [15], and clinical findings according to the H&H scale on admission were recorded.

Necessity of EVD insertion, modality of aneurysm obliteration, detection of cerebral vasospasm, frequencies of spasmolysis, delayed cerebral ischemia (DCI), decompressive craniectomy (DC), cerebrospinal fluid (CSF) shunt dependency, deep vein thrombosis (DVT), pulmonary embolism (PE), and severe cardiac events (SCE), i.e., incidents requiring electrical cardioversion or cardiopulmonary resuscitation, were recorded.

Survival at Days 14 and 30 after SAH was recorded. Follow-up at one year after the hemorrhage was recorded according to the Glasgow Outcome Scale (GOS) [16]. Favorable outcomes were defined as GOS 4 or 5.

### 2.2. Interdisciplinary Setting

Urgent EVD insertion was performed in cases with symptomatic hydrocephalus. Obliteration of the ruptured aneurysm was achieved within 24 h after admission. In all but one patient with a ruptured aneurysm of the middle cerebral artery (MCA), the aneurysm was clipped, while, in one patient with a ruptured aneurysm of the MCA, the aneurysm was coiled. In the remaining cases, an interdisciplinary decision as to the modality of aneurysm obliteration was made, and the ruptured aneurysm was treated accordingly. In cases with multiple aneurysms, sequence and modality of aneurysm obliteration were determined interdisciplinarily. All patients received neurosurgical intensive care.

### 2.3. Statistical Analysis

Statistical analysis was conducted using OpenOffice 4.1.3 and R 3.5.1 with R Studio 1.1.383 on a Mac OS X 10.14.4. Figures were created with R and R Studio. Chi square, Fisher’s exact, Welch’s t, and Wilcoxon rank sum served as statistical tests. To assess the impact of predictors on outcome variables, generalized linear models were fitted, and a proportional odds logistic regression was performed. Statistical significance was assumed with *p* values less than 0.05.

### 2.4. Ethical Approval

Upon our request in March 2018, the local ethics committee at the University Hospital Marburg considered an ethical approval unnecessary for this pseudonymized retrospective analysis.

## 3. Results

Mean age on admission was 56 years (range: 22–90 years). On admission, 63 patients (35.8%) were 60 years of age or older, and 9 patients (5.1%) were 80 years of age or older. One hundred six patients (60.2%) were female.

Overall, 167 of 176 patients (94.9%) presented with symptomatic hydrocephalus on admission and urgently received an EVD.

Clinical findings according to the H&H scale [12] on admission are given in Table 1. Information on blood distribution according to the modified Fisher scale in the initial CCT is provided in Table 2.

Clipped and coiled aneurysms by location are tabulated in Table 3. In 42 patients (23.9%), multiple cerebral aneurysms were detected. Ruptured aneurysms of the anterior circulation were significantly more often clipped, while ruptured aneurysms of the posterior circulation were significantly more often coiled (chi square test, *p* < 0.001).

Events of clinical significance during hospitalization are listed in Table 4. We found a significantly higher probability of DVT in patients who underwent clipping as opposed to coiling of ruptured cerebral aneurysms (Fisher’s exact test, *p* = 0.024).

Information on survival during the first month after the hemorrhage is provided in Table 5. At 30 days after the hemorrhage, we found a significantly higher probability of survival in patients who underwent clipping as opposed to coiling of ruptured cerebral aneurysms (generalized linear modeling, *p* = 0.0495). Outcome according to the GOS at one year after the hemorrhage is given in Table 6. Clinical data at 14 and 30 days after the hemorrhage were available in all patients, while follow-up at one year was obtained in 133 of 176 patients (75.6%).

**Figure 1:**
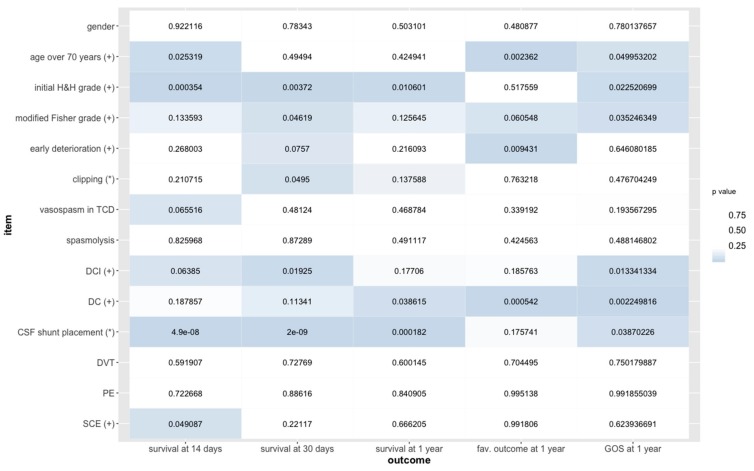
Statistical significance of potential predictors of outcome in 176 patients with aneurysmal subarachnoid hemorrhage as estimated from generalized linear models for dichotomous outcome variables (survival and fav. outcome) and proportional odds logistic regression for an ordinal outcome variable (GOS) after aneurysmal subarachnoid hemorrhage. Information on survival at Days 14 and 30 after the hemorrhage was available in all patients, while information on outcome at one year after the hemorrhage was obtained in 133 patients (75.6%). Abbreviations: H&H, Hunt and Hess; TCD, transcranial Doppler sonography; DCI, delayed cerebral ischemia; DC, decompressive craniectomy; CSF, cerebrospinal fluid; DVT, deep vein thrombosis; PE, pulmonary embolism; SCE, severe cardiac event; fav., favorable; GOS, Glasgow Outcome Scale; (*) true (dichotomous variables) or increasing (ordered values) values predictive of survival, favorable outcome, or high GOS score; (+) true (dichotomous variables) or increasing (ordered values) values predictive of mortality, unfavorable outcome or low GOS score.

The impact of potentially predictive variables on outcome after aSAH is illustrated in Figure 1.

## 4. Discussion

Various authors reported an initially poor H&H grade to predict an unfavorable outcome after aSAH [1,8,10,11,14]. This finding was confirmed in our study: according to our data, an initially poor H&H grade significantly predicted mortality at Days 14 and 30 after the hemorrhage, mortality at one year after the hemorrhage and a less favorable outcome according to the GOS at one year after the hemorrhage. We regularly refrain from obliterating ruptured cerebral aneurysms in patients who, prior to the onset of aSAH, have a history of severe cognitive impairment, such as progressive dementia, since we consider the potential benefit of aneurysm obliteration to these patients highly questionable.

Age as a predictor of outcome after aSAH has been reported before [10,13,14]. In our study, age of over 70 years on admission, being a cut-off age within the range of those of several other publications, was a strong predictor of mortality at 14 days after the hemorrhage, an unfavorable outcome and a less favorable outcome according to the GOS at one year after the hemorrhage. We share, however, the view of other authors that age alone should not be an objection as to the diagnosis and treatment of cerebrovascular diseases [17].

In other studies on aSAH, a high overall clot burden has been described as a predictive factor [18]. We found the extent of overall clot burden in the initial CCT, as recorded according to the modified Fisher scale, to be significantly associated with mortality at 30 days after the hemorrhage, and with a less favorable outcome according to the GOS at one year after the hemorrhage.

Clipping as opposed to coiling of ruptured aneurysms was a significant predictor of survival at 30 days after aSAH, but not later during the first year after the hemorrhage. As far as we know, a similar finding has not been reported in other studies before. We suppose that the reduction of overall clot burden, which is part of the standard clipping procedure as opposed to the standard coiling procedure after aSAH, leads to a temporary relief from spasmogenic stimuli in the subarachnoid space, which may explain our finding.

Furthermore, our single center experience has shown that particularly in patients with poor H&H grades, clipping of a ruptured aneurysm is often accompanied by DC in the same session. By contrast, in patients with coiled aneurysms usually a clinical deterioration somewhat later in the course of the disease gives rise to DC. The earlier onset of the effect of DC may lead to temporary recovery in clinically poor patients who undergo clipping of ruptured aneurysms, which may contribute to the statistically significant effect of clipping on survival at 30 days after the hemorrhage as observed in our study.

Coiling as opposed to clipping of ruptured aneurysms was significantly correlated with a lower frequency of DVT, while the frequency of PE did not significantly depend on the modality of aneurysm obliteration in our patients. One may assume that acetylsalicylic acid (ASS) and heparin in doses with therapeutic effects, as regularly administered after aneurysm coiling, help to prevent DVT, while larger patient cohorts need to be analyzed to prove a potential impact of ASS and heparin on the frequency of PE after aSAH.

The fact that aneurysms of the anterior circulation were significantly more often clipped, while aneurysms of the posterior circulation were significantly more often coiled, is primarily attributable to particularities of our interdisciplinary approach.

ED has been reported to predict an unfavorable outcome after aSAH [19], which was confirmed in our study: ED was significantly related to an unfavorable outcome at 12 months after the hemorrhage.

Other authors have reported SCE as well as elevated Troponin levels, electrocardiographic or echocardiographic abnormalities, to be linked to unfavorable outcomes after aSAH [20,21,22]. In our patients, SCE were a significant predictor of mortality at 14 days after the hemorrhage, but not later during the first year after the hemorrhage. This finding may become relevant when clinical decisions have to be consented with the patient’s next of kin: in patients with aSAH, a severely unstable cardiovascular situation during hospitalization after the hemorrhage occasionally tempts family members to fear lack of recovery and to demand intensive care not to be extended. Our study may provide a rationale to continue curative treatment in these cases.

We found that DCI was a significant predictor of mortality on Day 30 and of a less favorable overall outcome according to the GOS at one year after the hemorrhage, which is a finding other authors have reported before [14].

In our study, DC was a significant predictor of mortality, of an unfavorable outcome (i.e., GOS < 4) and of a less favorable outcome (i.e., a lower GOS) at one year after the hemorrhage. We found, however, that more than two out of three patients who required DC survived the first year after the hemorrhage. It therefore seems well warranted to indicate DC generously in patients who deteriorate due to malignant brain swelling after aSAH.

In our study, CSF shunt placement was a significant predictor of survival on days 14 and 30, and at one year after aSAH as well as of a less favorable outcome according to the GOS at one year after aSAH. This finding should be interpreted cautiously: of 97 patients who did not receive a CSF shunt, 14 (respectively, 17 and 21) patients were deceased at 14 (respectively, 30 and 365) days after the hemorrhage, while, of 79 patients who received a CSF shunt, 0 (respectively, 0 and 4) patients were deceased at 14 (respectively, 30 and 365) days after the hemorrhage. This observation is in accordance with the clinical experience that most patients with initially poor H&H grades simply do not survive long enough to receive a CSF shunt after aSAH.

### Limitations of Our Study

When interpreting our results, it should be kept in mind that this work is a single center retrospective study with an incomplete follow-up of 75.6% at one year after the hemorrhage. These facts set limitations to any generalized conclusion one would want to derive from our data. Our patients were treated at a university hospital in a country with an overall high standard of medical care.

## 5. Conclusions

SCE were predictive of mortality at 14 days after aSAH but not later during the first year after the hemorrhage.

Clipping as opposed to coiling of ruptured aneurysms was a significant predictor of survival at 30 days but not later during the first year after aSAH, while coiling as opposed to clipping of ruptured aneurysms was significantly related to a lower frequency of DVT during hospitalization.

Age over 70 years, H&H grade on admission, overall clot burden, ED, DCI, DC, and CSF shunt placement proved to be predictive of long-term outcome after aSAH.

Long-term results after clipping and coiling of ruptured aneurysms appear equal in an interdisciplinary setting that takes aneurysm localization, available staff, and equipment into account.

## Figures and Tables

**Table 1:** Clinical presentation according to the Hunt and Hess (H&H) scale [12] on admission in 176 patients with aneurysmal subarachnoid hemorrhage.

Initial H&H Grade	Number of Ruptured Aneurysms Clipped (*n* = 108)	Number of Ruptured Aneurysms Coiled (*n* = 68)	Statistical Test, *p*-Value
1	9	9	
2	27	16	
3	26	16	
4	29	17	
5	17	10	
			Wilcoxon rank sum, *p* = 0.14

**Table 2:** Blood distribution in the initial cranial computerized tomography (CCT) scan according to the modified Fisher scale [15] in 176 patients with aneurysmal subarachnoid hemorrhage.

Modified Fisher Grade	Number of Ruptured Aneurysms Clipped (*n* = 108)	Number of Ruptured Aneurysms Coiled (*n* = 68)
0	2	4
1	9	6
2	4	7
3	38	18
4	55	33

**Table 3:** Clipped and coiled aneurysms by location in 176 patients with aneurysmal subarachnoid hemorrhage.

Aneurysm Location	Number of Ruptured Aneurysms Clipped (*n* = 108)	Number of Ruptured Aneurysms Coiled (*n* = 68)	Statistical Test, *p-*Value
MCA ^1^	52	1	
ACA ^1^	11	4	
Acomm ^1^	27	26	
ICA paraophthalmic ^1^	3	4	
ICA supraophthalmic ^1^	13	12	
Pcomm ^1^	2	0	
SCA ^2^	0	1	
PICA ^2^	0	3	
Basilar artery ^2^	0	13	
Vertebral artery ^2^	0	4	
			
Ruptured aneurysms of the anterior circulation (subtotal of locations marked with superscript 1) *	108	47	
Ruptured aneurysms of the posterior circulation (subtotal of locations marked with superscript 2) *	0	21	
			chi square, *p* < 0.001

Abbreviations: MCA, middle cerebral artery; ACA, anterior cerebral artery; Acomm, anterior communicating artery; ICA, internal carotid artery; Pcomm, posterior communicating artery; SCA, superior cerebellar artery; PICA, posterior inferior cerebellar artery. Superscript 1, anterior circulation; superscript 2, posterior circulation. * Statistically significant finding.

**Table 4:** Events of clinical significance during hospitalization in 176 patients with aneurysmal subarachnoid hemorrhage.

Clinical Event	Number of Ruptured Aneurysms Clipped (*n* = 108)	Number of Ruptured Aneurysms Coiled (*n* = 68)	Statistical Test, *p*-Value
EVD insertion = yes	102	65	Fisher’s exact, *p* = 1
SCE = yes	5	7	Fisher’s exact, *p* = 0.218
Vasospasm in TCD = yes	39	16	chi square, *p* = 0.113
DCI = yes	49	34	chi square, *p* = 0.657
Spasmolysis = yes	5	4	Fisher’s exact, *p* = 0.736
DC performed = yes	29	13	chi square, *p* = 0.322
CSF shunt placed = yes	48	31	chi square, *p* = 1
DVT detected = yes *	8	0	Fisher’s exact, *p* = 0.024
PE detected = yes	4	1	Fisher’s exact, *p* = 0.65

Abbreviations: EVD, external ventricular drainage; SCE, severe cardiac event; TCD, transcranial Doppler sonography; DCI, delayed cerebral ischemia; DC, decompressive craniectomy; CSF, cerebrospinal fluid; DVT, deep vein thrombosis; PE, pulmonary embolism. * Statistically significant finding.

**Table 5:** Survival during the first month in 176 patients with aneurysmal subarachnoid hemorrhage.

Survival Status	Number of Ruptured Aneurysms Clipped (*n* = 108)	Number of Ruptured Aneurysms Coiled (*n* = 68)	Statistical Model, *p*-Value
Survived at 14 days	102	60	generalized linear modeling, *p* = 0.2107
Survived at 30 days *	101	58	generalized linear modeling, *p* = 0.0495

* Statistically significant finding.

**Table 6:** Outcome according to the Glasgow Outcome Scale (GOS) [16] at one year in 176 patients with aneurysmal subarachnoid hemorrhage.

GOS	Number of Ruptured Aneurysms Clipped (*n* = 108)	Number of Ruptured Aneurysms Coiled (*n* = 68)	Statistical Model, *p*-Value
1 at 1 year	14	11	
2 at 1 year	6	0	
3 at 1 year	25	11	
4 at 1 year	17	9	
5 at 1 year	24	16	
			proportional odds logistic regression, *p* = 0.4767
